# Does immune destruction drive all forms of bone marrow failure?

**DOI:** 10.1172/JCI161288

**Published:** 2022-08-01

**Authors:** Brian M. Dulmovits, Timothy S. Olson

**Affiliations:** 1 Cell Therapy and Transplant Section, Division of Oncology, Children’s Hospital of Philadelphia, Philadelphia, Pennsylvania, USA.; 2 Perelman School of Medicine, University of Pennsylvania, Philadelphia, Pennsylvania, USA.

## Abstract

Current paradigms of bone marrow failure (BMF) pathophysiology suggest that immune-mediated destruction of hematopoietic stem and progenitor cells (HSPCs) drives acquired aplastic anemia. In contrast, loss of HSPCs due to senescence and/or apoptosis causes BMF in inherited BMF syndromes. In this issue of the *JCI*, Casado and colleagues challenge this dichotomous conception by demonstrating that NK cell–dependent, immune-mediated hematopoietic suppression and HSPC clearance drive BMF in Fanconi anemia (FA). They show that genotoxic stress upregulates natural killer group 2 member D ligands (NKG2D-L) on FA HSPCs leading to NK cell cytotoxicity through NKG2D receptor activation. Inhibition of NKG2D–NKG2D-L interactions enhanced FA HSPC clonogenic potential and improved cytopenias in vivo. These results provide alternative targets for the development of immunosuppressive therapies to reduce HSPC loss and mitigate the risk of hematologic malignancies in FA.

## Bone marrow failure in Fanconi anemia

Fanconi anemia (FA) is the most common inherited bone marrow failure syndrome (IBMFS). FA is associated with congenital anomalies, predisposition to hematologic and nonhematologic malignancy, and aplastic bone marrow failure (BMF) developing in most cases by the second decade of life (1). Defective DNA repair resulting from germline mutations in approximately 23 genes underlies FA (2). Genotype-phenotype studies reveal that disease severity correlates with specific FA gene mutations (3).

**Figure 1:**
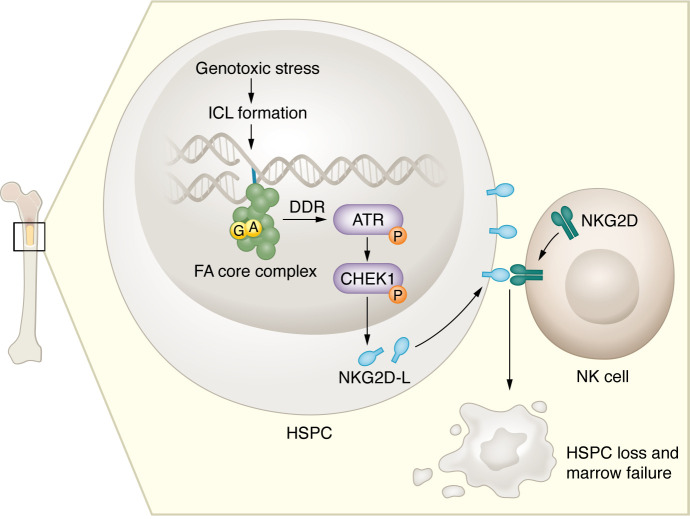
NKG2D/NKG2D-L interactions lead to NK cell–mediated loss of HSPCs in FA. Genotoxic stress promotes ICL formation, which results in the recruitment of FA/BRCA pathway members. In the setting of *FANC* mutations, DNA lesions remain unrepaired, causing activation of the DNA damage response (DDR) in HSPCs. Phosphorylation of ATR and CHEK1 stimulates the expression of NKG2D-Ls. NKG2D-Ls engage at the surface of HSPCs with NKG2D on NK cells, inducing HSPC apoptosis and subsequent bone marrow failure. Mutations in the FA core complex, including FANCA and FANCG protein mutations, were predominantly studied by Casado and colleagues (7).

FA genes encode proteins in the FA/breast cancer gene (BRCA) pathway that are required to correct DNA interstrand crosslinks (ICLs). DNA ICLs can be caused by ionizing radiation, alkylating chemotherapy, or endogenous aldehydes. ICL formation triggers the FA core complex to localize at DNA lesions, which then recruits other FA protein–containing complexes and ICL repair enzymes (4). Inactivating mutations in FA genes result in the inability to repair ICLs, leading to chromosomal instability. DNA damage in FA hematopoietic stem and progenitor cells (HSPCs) activates MYC and genotoxic stress/TP53 pathways, and induces aberrant inflammatory cytokine signaling (1, 5, 6). Through previously undefined downstream events, these changes drive HSPC loss and/or hematologic malignancies in FA. In this issue of the *JCI*, Casado et al. provide this missing downstream link by identifying an immune-mediated mechanism by which activation of DNA damage pathways causes BMF (7) (Figure 1).

## The NKG2D/NKG2D-L axis and cellular stress

Natural killer group 2 member D (NKG2D) is a C-type lectin-like receptor expressed by immune effector cells including NK cells and CD8^+^ T cells. NKG2D homodimers bind to the MHC class I chain-related gene and UL16 binding protein family ligands (i.e. NKG2D-Ls) expressed on target cells, inducing direct cytotoxicity and the production of hematopoiesis-suppressing IFN-/IL-2 (8). This process requires co-stimulatory activation (9). NKG2D–NKG2D-L interactions enable clearance of malignant, infected, or damaged cells. NKG2D-L transcriptional upregulation induced by DNA damage, reactive oxygen species, and heat shock protein responses is essential for cell removal (10, 11). NKG2D–NKG2D-L interactions may also facilitate NK cell education and tolerance (12).

Casado et al. demonstrated upregulation of NKG2D-Ls in primary cells from patients with FA and after knockdown of the Fanconi anemia complementation group A gene (*FANCA*) in HSPCs from healthy controls (HCs) (7). Treatment with crosslinking reagents mitomycin C (MMC) and formaldehyde further increased NKG2D-L expression in FA but not in HC cells. Upregulation of NKG2D-L was dependent on ATR/CHEK1 activation. Blockade of NKG2D–NKG2D-L interactions with monoclonal antibodies improved HSPC colony formation in vitro. This strategy also rescued anemia in an in vivo, mitomycin-induced BMF model using *Fanca^-/-^* mice (7).

These results suggest that NKG2D/NKG2D-L–mediated immune clearance by NK cells may be the downstream cause of BMF in FA. Despite long-described roles of cellular stress in FA and many other IBMFS, including Diamond Blackfan anemia (DBA), Schwachman Diamond syndrome, and telomere biology disorders (13), no studies have investigated NKG2D-L expression in these diseases. Only two reports have examined NKG2D-L upregulation in acquired BMF (14, 15).

## Bone marrow failure and the immune system

T cell–mediated destruction of HSPCs drives BMF in acquired aplastic anemia (aAA). Patients with aAA display reduced CD8^+^ T cell diversity, HSPC loss through CD8^+^ cell cytotoxicity, responsiveness of BMF to T cell–directed immune suppression therapy, inactivating HLA class I mutations, and somatic mutations within expanded T cell clonotypes (16–18). These data support CD8^+^ clones downstream of autoantigen recognition as the primary drivers of HSPC loss in aAA (19).

In contrast, no clear evidence supports CD8^+^ T cell and HLA class I responses as drivers of BMF in IBMFS. Indeed, most forms of T cell immunosuppression fail to induce remission in these diseases. Although glucocorticoids ameliorate some cytopenias in IBMFS, most notably the anemia in DBA, this response appears to occur independently of immune modulation (20). This lack of clinical efficacy has dissuaded study of immune-mediated mechanisms in IBMFS until now. Casado et al. demonstrate that though HLA class I–restricted CD8^+^ T cell immune responses may differ from those in aAA, NK cell immune mechanisms may instead serve as the likely drivers of BMF in IBMFS (7). These responses require further study.

## Conclusions and future directions

The only proven cure for BMF in FA is allogeneic stem cell transplantation (alloSCT). AlloSCT outcomes are limited by suitable donor availability and morbidity due to graft-versus-host disease and conditioning-related toxicity (21). Unconditioned, autologous stem cell–based gene therapy for FA is in development but is often limited by low numbers of surviving stem cells at the time of HSPC collection (22). Therapies that forestall HSPC loss in FA, enabling allogeneic donor identification or sufficient autologous HSPC collection, are critically needed. Androgen therapy slows worsening cytopenias in some (but not all) patients with FA. However, the impact of androgen therapy on HSPC loss is less certain (23).

The Casado et al. study signals a conceptual paradigm shift in BMF pathophysiology related to FA (7). The study defines the need for additional testing of therapeutics that inhibit NKG2D–NKG2D-L interactions to block immune-mediated HSPC clearance, an approach that could buy critical time for FA patients pursuing autologous gene therapy or alloSCT. These investigations should also clarify whether subsets of NKG2D-expressing CD8^+^ T cells also play roles in HSPC clearance, providing alternative immunosuppression targets. Finally, NKG2D–NKG2DL interactions may promote HSPC loss in other IBMFS, since HSPC stress represents a shared upstream pathophysiology. Thus, these findings should promote research and therapeutics across the BMF field.

## Version 1 08/01/2022

Electronic publication
